# Successful Treatment of Severe Plaque Psoriasis in an Adolescent With a Combination of Methotrexate and Acitretin: A Case Report

**DOI:** 10.7759/cureus.96546

**Published:** 2025-11-11

**Authors:** Mehmet Ali Yildirim

**Affiliations:** 1 Dermatology, Denizli State Hospital, Denizli, TUR

**Keywords:** acitretin, adolescent, methotrexate, psoriasis, stigma

## Abstract

Psoriasis is a chronic inflammatory skin disease that can occur at any age. It is a significant disease due to its appearance in visible areas, its potential to persist for years without treatment, and its association with various comorbidities. Severe psoriasis in children and young adolescents is particularly important because this period of life has a crucial impact on psychological development. The case presents a young adolescent with severe plaque psoriasis who was successfully treated with a combination of methotrexate and acitretin. This combination is not routinely used in daily practice due to concerns that it may cause serious hepatotoxicity.

## Introduction

Plaque psoriasis typically presents as sharply demarcated squamous papules and plaques. Although guttate psoriasis, associated with *streptococcal* infections, is much more common in the pediatric population than in adults, plaque psoriasis remains the most common clinical form during this period. In nearly one-third of all psoriasis patients, the disease begins before the age of 18 [1]. Research shows that psoriasis beginning in childhood significantly affects children's psychological health and increases the risk of depression, anxiety, suicidal tendencies, substance abuse, and eating disorders. Psoriasis can also lead to stigmatization, especially in young people [2,3]. As in adults, psoriasis in this stage of life is known to be associated with comorbidities such as obesity, dyslipidemia, metabolic syndrome, and tobacco and drug addiction. The onset of these comorbidities at an early age can lead to persistent health problems in adulthood [4,5]. Today, almost all agents used in the treatment of psoriasis have been developed based on trials conducted on adults, and the lack of consensus in international treatment guidelines for the pediatric population also poses a problem [6]. For these reasons, young patients with severe psoriasis in particular require close monitoring and effective treatment.

## Case presentation

**Figure 1:**
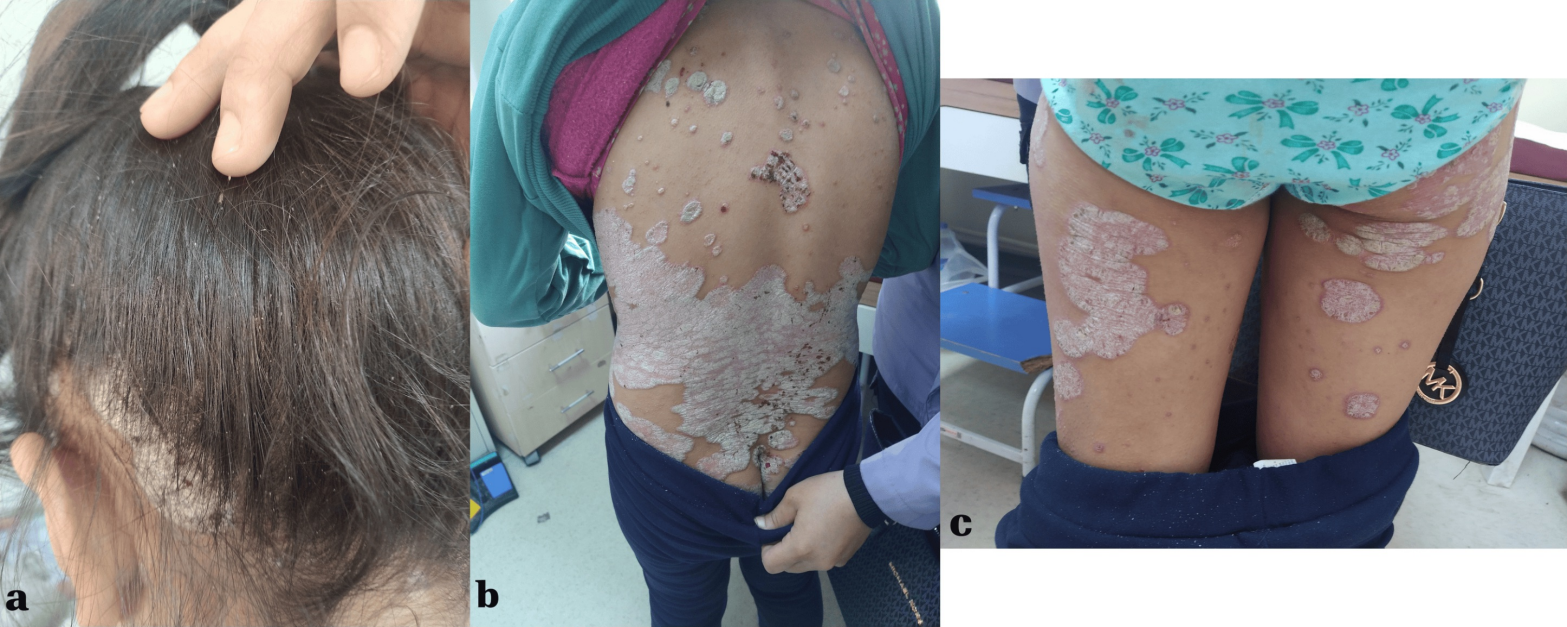
Thick, well-demarcated papules and plaques with silvery-white scales on her scalp (a), back (b), and lower extremities (c)

An 11-year-old girl presented with complaints of scaly lesions covering her body. Dermatological examination revealed widespread, sharply demarcated, indurated, scaly plaque lesions covering almost the entire scalp, nape of the neck, forehead, trunk, and extremities (Figure 1).

**Figure 2:**
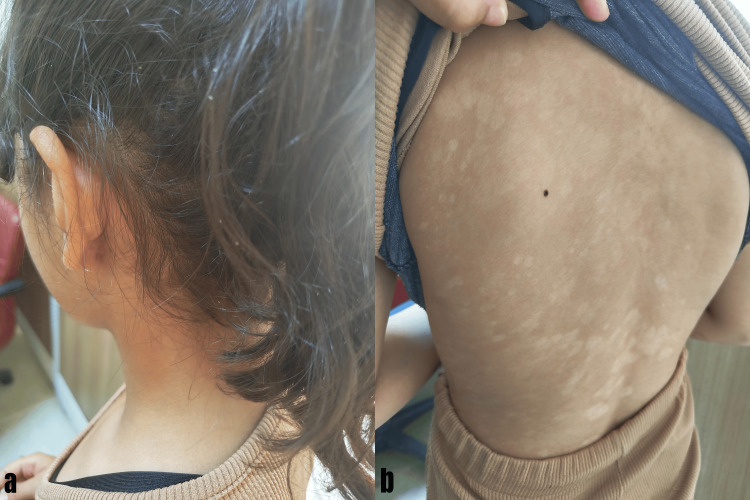
After eight weeks of treatment, a lesion-free scalp (a), healed lesions with post-inflammatory hypopigmentation on the patient's back (b)

Patient clinically diagnosed with plaque psoriasis. It was learned that she had had the disease for two years, had previously undergone topical treatments only, and that the lesions had never completely disappeared. Also, it's learned that since the onset of her illness, she has withdrawn into herself, refusing to apply creams because they don't work, and her friends at school keep their distance from her, afraid of the disease being contagious. There were no additional illnesses, and the lab results were within normal limits. The Psoriasis Area Severity Index (PASI) was 35. Although phototherapy and biological agents are relatively safe, conventional treatments were preferred because these therapies are not available at the secondary care public hospital in the rural area. Due to psoriasis's significant negative impact on the patient's quality of life and its clinical severity, it was decided to use a combination of acitretin, which acts by regulating keratinocyte proliferation, and methotrexate, which suppresses inflammation. However, since hepatotoxicity may increase, both drugs were started at a lower dose than when used alone (5 mg/day of acitretin and 7.5 mg/week of methotrexate; the patient was 30 kg). The family was informed about the treatment plan and possible side effects. At the follow-up appointment two weeks later, there was very little improvement in her clinic, and laboratory results were normal. At her eight-week follow-up appointment, she was smiling and happy. It was observed that nearly all of the lesions on her body had healed with post-inflammatory hypopigmentation (Figure 2).

PASI was three, and laboratory tests were within normal limits. The patient underwent monthly clinical and laboratory follow-ups. No side effects were reported other than nausea lasting 1-2 days after methotrexate injection and occasional fatigue. Laboratory results remained within normal limits. For rare mild flare-ups, topical calcipotriol-betamethasone dipropionate combination was added to the treatment. Acitretin was discontinued after five months. Approximately one year later, methotrexate was gradually reduced over two months and discontinued. PASI did not exceed five during the follow-up period.

## Discussion

As dermatologists, we mostly encounter severe psoriasis in adults. It's possible that we may lack sufficient experience in the use of both conventional medications and biological treatments in children, or we can be even more concerned about the side effects, especially in pediatric patients. For these reasons, some pediatric psoriasis patients are unable to receive adequate and appropriate treatment. However, it should not be forgotten that chronic skin diseases seen in childhood and early adolescence have a significant impact on the patient's quality of life and mental health, and psoriasis ranks among the top of these diseases [2,3]. The patient presented here also exhibited unhappiness, social withdrawal, and stigmatization. Psoriasis, when not adequately treated, is a major problem, as the chronic inflammation lasting for years causes comorbidities such as psoriatic arthritis, metabolic syndrome, and cardiovascular diseases [4,5]. Phototherapy and acitretin combination may be a good first option for the patient presented, but phototherapy was not available at the hospital. Biological agents are effective and appear to be safe. However, controlled studies involving long-term follow-up of their use, especially in childhood, are insufficient [7]. In my country, for these medications to be covered by insurance, the hospital must be a tertiary care facility, and the patient must have previously undergone phototherapy or conventional treatment. Due to the severity of the patient's clinical condition and the negative impact on her quality of life, adequate treatment was needed. The available options were acitretin and methotrexate, and these two drugs were used together to achieve a better response than when used alone. As is known, one regulates keratinocyte proliferation while the other affects inflammatory processes; these two effects together produce a synergistic response. When used alone, the recommended dosage for these drugs in the pediatric population is 0.2 to 0.7 mg/kg/week for methotrexate and 0.5 to 1 mg/kg/day for acitretin, which is similar to the dosage for adults [8]. In this case, the drugs were used at a much lower dose than these due to concern of hepatotoxicity (5 mg/day of acitretin and 7.5 mg/week of methotrexate). Folic acid replacement was not administered in order to avoid further reducing the effect of low-dose methotrexate. However, it has also been suggested that folic acid supplementation may reduce hepatotoxicity [9]. In the case presented, no deterioration in liver function tests or decrease in white blood cell count was observed during the patient's monthly follow-ups. Serum procollagen III amino-terminal peptide, a more sensitive marker of hepatotoxicity, could not be evaluated because it could not be tested in the hospital laboratory. Since near-complete remission was achieved in a short period of time and this remission was seen to be sustained, systemic treatment was discontinued (acitretin 5 months later, methotrexate approximately one year). There are studies in the literature on the combined use of methotrexate and acitretin in the treatment of psoriasis, and these studies show that better and longer-lasting responses are achieved compared to the use of these drugs alone. In addition, it is emphasized that the combined use of these drugs at lower than normal doses is safer in terms of the risk of hepatotoxicity [10,11].

## Conclusions

While biological treatments developed in recent years have made treating psoriasis easier, access to these drugs can sometimes be limited. This case is important because it shows that well-known and cost-effective traditional treatments, such as methotrexate and acitretin, which have been used for decades, can be combined and produce good results at lower doses. The case is also important because it shows how a serious skin disease can affect a child's life and how treating this disease appropriately can ensure the child's overall well-being.
